# Cytokine Production in Response to Soluble *Leishmania Aethiopica* Antigen by Whole Blood Cells from Patients with Different Clinical Presentations of Cutaneous Leishmaniasis

**DOI:** 10.4269/ajtmh.24-0581

**Published:** 2025-02-11

**Authors:** Bizuayehu Gashaw, Endalew Yizengaw, Edward Cruz Cervera, Endalkachew Nibret, Dessalegn Tamiru, Ingrid Müller, James A. Cotton, Yegnasew Takele, Pascale Kropf

**Affiliations:** ^1^ Amhara Public Health Institute, Bahir Dar, Ethiopia;; ^2^ Department of Biology, College of Science, Bahir Dar University, Bahir Dar, Ethiopia;; ^3^ Department of Medical Laboratory Science, College of Medicine and Health Sciences, Bahir Dar University, Bahir Dar, Ethiopia;; ^4^ Institute of Biotechnology, Bahir Dar University, Bahir Dar, Ethiopia;; ^5^ Department of Infectious Disease, Imperial College London, London, United Kingdom;; ^6^ Nefas Mewcha Hospital, Lay Gayint, Ethiopia;; ^7^ School of Biodiversity, One Health and Veterinary Medicine, College of Medical, Veterinary and Life Sciences, University of Glasgow, Glasgow, United Kingdom

## Abstract

Cutaneous leishmaniasis (CL), a parasitic disease caused by *Leishmania aethiopica*, is a major health problem in Ethiopia. It presents mostly as three different clinical forms: localized CL characterized by small lesions that ulcerate; diffuse CL defined by multiple nonulcerating nodules; and mucocutaneous leishmaniasis, where the mucosa of the nose or the mouth is affected. The mechanisms resulting in the development of these different clinical presentation are still poorly understood. Here, we recruited a cohort of CL patients presenting with different forms of CL in northwest Ethiopia as well as cohort of healthy nonendemic controls. We assessed the capacity of whole blood cells from these cohorts to produce cytokines in response to soluble *L. aethiopica* antigen and compared these levels between the different clinical presentations of CL and healthy nonendemic controls. Our results show that the levels of antigen-specific cytokines produced by whole blood cells from CL patients were higher as compared with controls. However, these cytokine levels were similar among the different clinical presentations. In conclusion, the results of our study indicate that variations in clinical manifestations of CL are not associated with differences in antigen-specific cytokine profiles.

## INTRODUCTION

Cutaneous leishmaniasis (CL) is a serious health problem in Ethiopia, where it is mainly caused by *Leishmania aethiopica* parasites.^1^ In Ethiopia, it presents mostly as three different clinical forms. The most common form of CL is localized cutaneous leishmaniasis (LCL), which is characterized by small lesions that ulcerate. Cutaneous leishmaniasis can also present as diffuse cutaneous leishmaniasis (DCL), which is characterized by multiple nonulcerating nodules, and mucocutaneous leishmaniasis (MCL), where the mucosa of the nose or the mouth is affected. We have recently investigated the epidemiology of a focus of CL in Nefas Mewcha, northwest Ethiopia and showed that LCL can be further divided in at least two forms: contained localized cutaneous leishmaniasis (C LCL) when the lesions present with a distinct border and spreading localized cutaneous leishmaniasis (S LCL) characterized by lesions without clear edges.^2^

Localized cutaneous leishmaniasis usually heals spontaneously; however, some forms are persistent and require treatment. Diffuse cutaneous leishmaniasis and MCL are difficult to treat and relapse frequently. Cutaneous leishmaniasis often leave disfiguring scars that result in societal stigmatization. The mechanisms resulting in the development of these different forms of CL are still not understood. We have recently shown that no individual parasite genetic variants were significantly associated with disease presentation and that plasma levels of cytokines and chemokines were similar between all CL presentations.^1^

In the present study, we tested whether differences in antigen-specific cytokine levels as produced by whole blood cells might be associated with the different CL presentations.

## MATERIALS AND METHODS

### Patient recruitment.

For this study, we recruited 60 CL patients who were part of the cohort described in the work by Yizengaw et al.^2^ Patient recruitment, diagnosis, and characteristics are also described in the work by Yizengaw et al.^2^ Parasite grading was assessed as described in the work by Chulay and Bryceson.^3^ In addition, 10 healthy nonendemic controls (HNECs) were recruited from the staff of Imperial College London, United Kingdom.

### Blood sample collection.

Four milliliters of venous blood was drawn in heparin tubes. One milliliter was centrifuged, and the plasma was collected and immediately frozen to be used at a later time point to measure the levels of cytokines. Three milliliters of blood was used for the whole blood assay as described below.

### Whole blood assay.

Soluble *Leishmania* antigen (SLA) was prepared as described in the work by Adem et al.^4^ from *L. aethiopica* isolated from three different LCL patients. Three milliliters of blood collected in heparinized tubes was used, and 1-mL aliquots were distributed in three tubes and stimulated with SLA added at a concentration of 5 µg/mL and phytohaemagglutinin (PHA; Sigma-Aldrich, Burlington, MA) at 10 µg/mL. Unstimulated blood was used as a negative control. Supernatants were collected after 24 hours of incubation at 37°C and stored at −20°C for further analysis.

The cytokine concentrations obtained with the unstimulated whole blood cells were subtracted from the cytokine concentrations obtained with PHA or SLA.

### Cytokine measurements.

Interferon gamma (IFNγ), interleukin-2 (IL-2), IL-4, IL-6, IL-10, IL-12p70, IL-1β, IL-13, and tumor necrosis factor alpha (TNFα) were measured in plasma and supernatants collected as described above by multiplex assay using a cytokine panel V-PLEX Kit following the manufacturer’s instructions (Meso Scale Diagnostics, Gaithersburg, MD). This technique enables the simultaneous detection of multiple cytokines in a single well by using electrochemiluminescence technology.

## STATISTICAL ANALYSES

Data were evaluated for statistical differences as specified in the tables and figures using Mann–Whitney and Kruskal–Wallis tests. Differences were considered statistically significant at *P* <0.05. Unless otherwise stated, summary statistics given are medians followed by interquartile ranges.

## RESULTS

### Characterization of the cohort.

The cohort consisted of 60 CL patients (Table 1). Forty-three CL patients presented with LCL; of those patients, 31 presented with C LCL, and 12 presented with S LCL. Seventeen CL patients presented with MCL. In agreement with our previous study,^2^ there were more males than females (Table 1), and there were no significant differences in their age, body mass index (BMI) (Table 2), duration of illness, and parasite grading (Table 3). In line with our previous data,^1^ only IFNγ and TNFα were detectable in the plasma of CL patients, and no significant differences in the levels of these two cytokines were observed between the three different clinical presentations and HNECs (Supplemental Table 1).

**Table 1:** Cutaneous leishmaniasis patients with different clinical presentations (*N* = 60)

Sex	C LCL	S LCL	MCL
Females (*n* = 22)	13	6	3
Males (*n* = 38)	18	6	14

C LCL = contained localized cutaneous leishmaniasis; MCL = mucocutaneous leishmaniasis; S LCL = spreading localized cutaneous leishmaniasis.

**Table 2:** Age and body mass index of cutaneous leishmaniasis patients with different clinical presentations (*N* = 60)

Variable	C LCL (*n* = 31)	S LCL (*n* = 12)	MCL (*n* = 17)	*P*-Value*
Age, years	35 (25–50)	37 (21–56)	22 (18–36)	0.1064
BMI, kg/m^2^	21 (19–23)	21 (19–23)	20 (19–21)	0.1785

BMI = body mass index; C LCL = contained localized cutaneous leishmaniasis; MCL = mucocutaneous leishmaniasis; S LCL = spreading localized cutaneous leishmaniasis. BMI was measured by dividing weight (kilograms) by the square of the height (meters).

* Statistical differences were measured by the Kruskal–Wallis test.

**Table 3:** Duration of illness and parasite grading of cutaneous leishmaniasis patients with different clinical presentations

Variable	C LCL	S LCL	MCL	*P*-Value*
Duration of illness, months (*n* = 60)	7 [4–12] (*n* = 31)	12 [9–12] (*n* = 12)	9 [7–12] (*n* = 17)	0.1905
Parasite grading, + (*n* = 47)	1 [1–2] (*n* = 23)	1 [1–2] (*n* = 11)	1 [1–2] (*n* = 13)	0.9567

C LCL = contained localized cutaneous leishmaniasis; MCL = mucocutaneous leishmaniasis; S LCL = spreading localized cutaneous leishmaniasis. The parasite grading was performed on 47 patients as described in the Materials and Methods section.

* Statistical differences were measured by the Kruskal–Wallis test.

### Whole blood assay.

**Figure 1.**
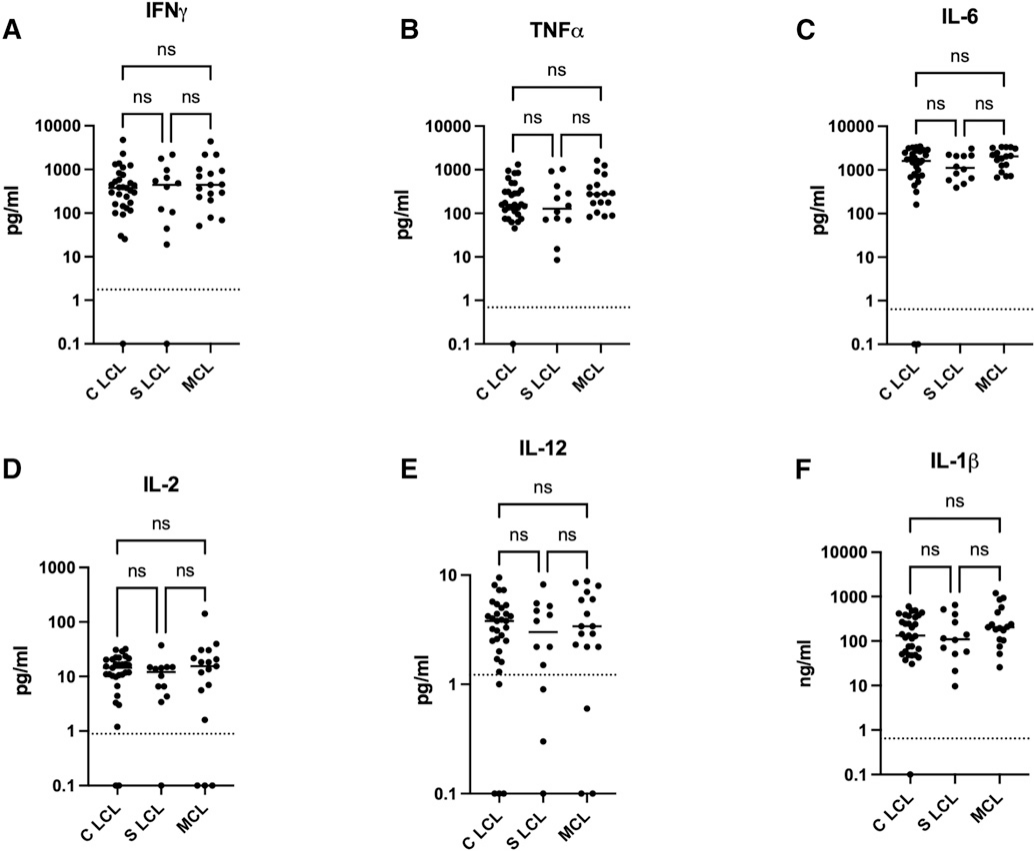
Levels of antigen-specific proinflammatory cytokines produced by whole blood cells from contained localized cutaneous leishmaniasis (C LCL), spreading localized cutaneous leishmaniasis (S LCL), and mucocutaneous leishmaniasis (MCL) patients are shown. One milliliter of whole blood each from 31 C LCL, 12 S LCL, and 17 MCL patients was incubated with soluble *Leishmania* antigen (SLA) at 37°C as described in the Materials and Methods section, and a further 1 mL of unstimulated blood was used as a negative control. Supernatants were collected after 24 hours of incubation at 37°C. The cytokine concentrations of (**A**) IFNγ, (**B**) TNFα, (**C**) IL-6, (**D**) IL-2, (**E**) IL-12, and (**F**) IL-1β were measured by multiplex assay as described in the Materials and Methods section. The concentrations obtained with the unstimulated whole blood cells were subtracted from the cytokine concentrations obtained with SLA. Statistical differences were measured by the Kruskal–Wallis test. IFN = interferon; IL = interleukin; ns = not significant; TNF = tumor necrosis factor.

**Figure 2.**
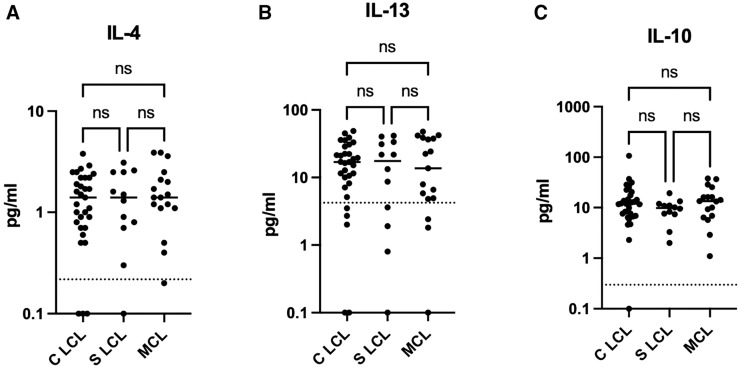
Levels of antigen-specific anti-inflammatory and regulatory cytokines produced by whole blood cells from contained localized cutaneous leishmaniasis (C LCL), spreading localized cutaneous leishmaniasis (S LCL), and mucocutaneous leishmaniasis (MCL) patients are shown. One milliliter of whole blood each from 31 C LCL, 12 S LCL, and 17 MCL patients was incubated with soluble *Leishmania* antigen (SLA) at 37°C as described in the Materials and Methods section, and a further 1 mL of unstimulated blood was used as a negative control. Supernatants were collected after 24 hours of incubation at 37°C. The cytokine concentrations of (**A**) IL-4, (**B**) IL-13, and (**C**) IL-10 were measured by multiplex assay as described in the Materials and Methods section. The concentrations obtained with the unstimulated whole blood cells were subtracted from the cytokine concentrations obtained with SLA. Statistical differences were measured by the Kruskal–Wallis test. IL = interleukin; ns = not significant.

Next, we assessed whether the different clinical presentations were associated with different levels of cytokines as measured in the supernatants of whole blood cells cultured in the presence of SLA and PHA. All of the levels of cytokines were significantly higher when the whole blood cells were activated with SLA or PHA (data not shown) as compared with unstimulated whole blood cells. Results presented in Figure 1 and Supplemental Table 2 show that the levels of pro- and anti-inflammatory cytokines (Figure 2A and B) and regulatory cytokines (Figure 2C) were similar among the different clinical presentations. Of note, the levels of all cytokines were higher in CL patients as compared with HNECs; however, this difference was not significant for IL-2 (Supplemental Table 3).

## DISCUSSION

Here, we show that all cytokines measured were detectable in the supernatants of whole blood cells incubated with SLA but that there were no differences in cytokine concentration between the different clinical forms of CL. Our results also show that the levels of these cytokines were higher in CL patients as compared with HNECs.

Whereas the whole blood assay is widely used to measure antigen-specific cytokines, such as IFNγ and IL-10 in patients with visceral leishmaniasis,^5^^,^^6^ little is known about this assay in CL patients. A study by Mohammadi et al.^7^ measured detectable levels of IFNγ, IL-5, and IL-10 in the supernatants of SLA-stimulated whole blood cells isolated from patients infected with *Leishmania major* or *Leishmania tropica*. Another study showed that after activation with SLA, whole blood cells from patients with *Leishmania braziliensis* infections produced higher levels of IFNγ, chemokine (C-X-C motif) ligand 9 (CXCL9), CXCL10, and chemokine (C-C motif) ligand 2 (CCL2) as compared with unstimulated whole blood cells.^8^ Although cytokines, such as IFNγ, IL-2, IL-4, IL-6, IL-10, and IL-13, can be produced by T cells,^9^ it has been shown that other cells can also produce some of these cytokines. For example, monocytes have the ability to produce IFNγ,^10^ TNFα,^11^ IL-6,^12^ IL-12,^13^ and IL-10.^14^ It is, therefore, possible that in the present study, some of the cytokines were produced by both antigen-specific cells and other cells, such as monocytes. Indeed, *L. aethiopica*-soluble antigen has been shown to activate both monocytes and neutrophils in whole blood from CL patients.^15^ This might be the result of direct activation by SLA or indirect activation through cytokines produced by antigen-specific cells.

## CONCLUSION

Cutaneous leishmaniasis is not characterized by polarized Th1 and Th2 responses but rather, by a mixed cytokine profile.^16^^–^^19^ Indeed, in our study, we show that the levels of pro- and anti-inflammatory cytokines as well as regulatory cytokines were similar in all three forms of CL. However, only a small number of patients were tested, and they had different ages, duration of illness, numbers of lesions, and BMI. A larger cohort will be needed to assess whether an immune signature as measured by using whole blood cells activated with SLA is associated with different clinical presentation.

## Supplemental Materials

10.4269/ajtmh.24-0581 Supplemental Materials
