# Supplementary material for: Cytokine Production in Response to Soluble Leishmania Aethiopica Antigen by Whole Blood Cells from Patients with Different Clinical Presentations of Cutaneous Leishmaniasis
Source: Am J Trop Med Hyg. 2025 Feb 11;112(4):749–52. doi: 10.4269/ajtmh.24-0581 (PMC11965761; doi:10.4269/ajtmh.24-0581)
Supplement: Supplemental Materials [file tpmd240581.SD1.pdf]

Table S1: Comparison of cytokine levels in the plasma from C LCL, S LCL, MCL patients and healthy nonendemic controls

	**C LCL**	**S LCL**	**MCL**	**HNEC**	***p values**
**IFNγ (pg/ml)**	6.1 [4.4-13.5]	6.0 [4.3-8.4]	6.2 [4.4-8.2]	6.5 [3.4-8.5	0.8292
**TNFα (pg/ml)**	1.0 [0.7-1.2	0.8 [0.5-1.3]	0.7 [0.5-0.9]	0.7 [0.5-1.0]	0.0909

IFNγ and TNFα levels were measured in the plasma isolated from the blood of C LCL (n=23), S LCL (n=12), MCL (n=16) and healthy nonendemic controls (HNEC, n=11) by multiplex assay, as described in Materials and Methods.

*Statistical differences were measured by Kruskal-Wallis

Table S2: Comparison of cytokine levels in the supernatants of whole blood cells from C LCL, S LCL and MCL patients stimulated with SLA

	**C LCL**	**S LCL**	**MCL**	***p values**
**IFNγ (pg/ml)**	379 [145-753]	443 [60-888]	444 [216-969]	0.7673
**TNFα (pg/ml)**	158 [95-344]	128 [70-386]	275 [138-617]	0.2262
**IL-6 (pg/ml)**	1609 [705-2647]	1114 [596-2124]	2034 [1076-3156]	0.2212
**IL-2 (pg/ml)**	14 [10-21]	12 [5-15]	15 [4-26]	0.4547
**IL-12 (pg/ml)**	3.8 [2-5]	3 [1.1-5.1]	3.4 [2.2-6.5]	0.7802
**IL-1β (pg/ml)**	132 [51-366]	110 [53-367]	205 [106-506]	0.3462
**IL-4 (pg/ml)**	1.4 [0.7-2.2]	1.4 [0.7-2.5]	1.4 [1.1-2.3]	0.7779
**IL-13 (pg/ml)**	16.9 [8.2-28.8]	17.6 [2.3-33]	13.7 [4.9-38.3]	0.9596
**IL-10 (pg/ml)**	12 [6.9-20.3]	9.8 [7.5-11.7]	13.4 [6.7-20.9]	0.3086

1 ml of whole blood from 31 C LCL, 12 S LCL and 17 MCL patients was incubated with SLA at 37°C as described in Materials and Methods. After 24 hrs, the supernatants were collected and the cytokine levels were measured in the supernatants by multiplex assay, as described in Materials and Methods.
*Statistical differences were measured by Kruskal-Wallis.

Table S3: Comparison of cytokine levels in the supernatants of whole blood cells stimulated with SLA between CL patients and HNEC

	**CL**	**HNEC**	**^p values**
**IFNγ (pg/ml)**	391 [134-824]	143 [101-237]	0.0143
**TNFα (pg/ml)**	176 [90-414]	17.6 [0.1-85.3]	<0.0001
**IL-6 (pg/ml)**	1652 [729-2474]	452 [191-786]	0.0003
**IL-2 (pg/ml)**	14.1 [6.6-20.5]	13.7 [9-16.5]	0.8589
**IL-12 (pg/ml)**	3.4 [2.1-5.3]	1.5 [0.6-2.1]	0.0018
**IL-1β (pg/ml)**	158 [60-377]	40 [35-92]	0.0014
**IL-4 (pg/ml)**	1.4 [1-2]	0.4 [0.1-1.0]	0.0013
**IL-13 (pg/ml)**	16.7 [5.3-32.8]	1.6 [0.6-2.1]	<0.0001
**IL-10 (pg/ml)**	11.8 [7.0-16.2]	6.8 [4.4-11.3]	0.0470

1 ml of whole blood from 60 LCL patients and 10 healthy non-endemic controls was incubated with SLA at 37°C as described in Materials and Methods. After 24 hrs, the supernatants were collected and the cytokine levels were measured in the supernatants by multiplex assay, as described in Materials and Methods.
^Statistical differences were measured by Mann-Whitney.
